# Insect biomass density: measurement of seasonal and daily variations using an entomological optical sensor

**DOI:** 10.1007/s00340-023-07973-5

**Published:** 2023-01-17

**Authors:** Adrien P. Genoud, Topu Saha, Gregory M. Williams, Benjamin P. Thomas

**Affiliations:** 1 Department of Physics, New Jersey Institute of Technology, Newark, NJ USA; 2 Center for Vector Biology, Rutgers University, New Brunswick, NJ USA

## Abstract

Insects are major actors in Earth’s ecosystems and their recent decline in abundance and diversity is alarming. The monitoring of insects is paramount to understand the cause of this decline and guide conservation policies. In this contribution, an infrared laser-based system is used to remotely monitor the biomass density of flying insects in the wild. By measuring the optical extinction caused by insects crossing the 36-m long laser beam, the Entomological Bistatic Optical Sensor System used in this study can evaluate the mass of each specimen. At the field location, between July and December 2021, the instrument made a total of 262,870 observations of insects for which the average dry mass was 17.1 mg and the median 3.4 mg. The daily average mass of flying insects per meter cube of air at the field location has been retrieved throughout the season and ranged between near 0 to 1.2 mg/m^3^. Thanks to its temporal resolution in the minute range, daily variations of biomass density have been observed as well. These measurements show daily activity patterns changing with the season, as large increases in biomass density were evident around sunset and sunrise during Summer but not during Fall.

## Introduction

The decline of insects in terms of numbers and species diversity has become a major concern both in the scientific community and to the general public [1–4]. While there is much variation across species, space and time, multiple studies point toward a rate of decline in abundance in the range of 1 to 2% per year [4–7]. Well-documented pollinators, such as bumble bees (*Bombus sp.*) have shown evidence of an ongoing decline in diversity [4, 8–10]. Similarly, the European Butterfly Indicator for Grassland species points toward a near 50% decline in butterflies between 1990 and 2011 in Europe [11], while Hallmann et al. [4] observed a 75% decline in insect biomass in Germany between 1989 and 2016. Overall, the stressors identified as responsible for this decline appear to be very diverse: agricultural intensification, land-use change, deforestation, habitat fragmentation, the use of pesticides, pollution, and climate change [3, 12–14].

However, direct evidence of a geographically widespread decline of most groups of insects remains sparse: population trends, when available, show great variance between insect families or groups. For example, terrestrial insects seem to be more at risk than freshwater insects, of which abundance is increasing in some cases [15]. Similarly, studies on the effect of short-term events on insect populations, like weather events or human interventions, suffer from the same lack of data. Insect activities and abundance over such a short-time scale are generally unavailable as traditional methods only have the ability to monitor the population over days or weeks, not hours. Lack of reliable data on insect populations is now considered a significant issue in the field of entomology [16, 17]. Indeed, better tools are necessary to understand complex insect behaviors and the causes of the observed decline in insect biomass.

Monitoring the population of flying insects and its biomass is generally done through the use of interception traps, such as malaise traps [18, 19], or attractant traps using light, pheromones, food, or CO_2_ as bait [20–25]. While those methodologies provide high accuracy for the identification of the family, genus, species, and sex of the captured insects through expert identification or DNA barcoding [26, 27], they do present some disadvantages. While there has been an improvement in automated and bulk analysis with the use of smart traps [28, 29] or automated insect barcoding [30], most traps still require long and/or expensive laboratory analysis where insects must be identified and weighed. Entomologists still face a lack of large-scale and long-term data on biomass trends. Furthermore, traps are based on a destructive sampling process where insects are killed, which may be problematic both in terms of impact on potentially endangered species but also in terms of skewing abundance measurements when a large number of insects are removed from the ecosystem. Additionally, the attractive range of the traps is generally unknown and changes with weather conditions [31–34]. As a consequence, traps provide poor population or biomass density estimates because the area of effect of the trap is usually unknown. Baits used to attract insects are not evenly effective across species, age, and sex groups, resulting in systematic errors between species present near traps [35–38]. Finally, traps suffer from poor time resolution: due to the time required to identify captured insects, traps are often used sporadically, e.g., 1 day per week in the six-year campaign reported by Kampen et al. [39]. Even while they operate, the time at which each insect has been captured is generally unknown, although physical traps with bottle rotator systems provide some information about the time of capture, the temporal resolution remains low.

In the meantime, entomological optical systems based on light-matter interaction have seen significant improvement over the last two decades [40–46] and could potentially complement trapping techniques and unlock some of the limitation of the field by providing biomass estimation and, in some cases, even the identification of the flying specimen [47–49]. In this contribution, we present the results of an Entomological Bistatic Optical Sensor System (eBoss) that allows for the continuous monitoring of the flying insect volumetric biomass density, expressed in mg/m^3^. This metric, referred to simply as biomass density, describes the mass of an insect flying through one cubic meter of air at any given time. This study follows an earlier experiment [50], undertaken at the same location but using a different optoelectronic technology (based on backscattering rather than extinction). As the focus of the present study is an estimation of biomass density, and this was not attempted in the previous investigation, a direct comparison of the two methods is not possible.

## Methods and materials

### Experimental setup

#### Entomological bistatic optical sensor system

**Fig. 1:**
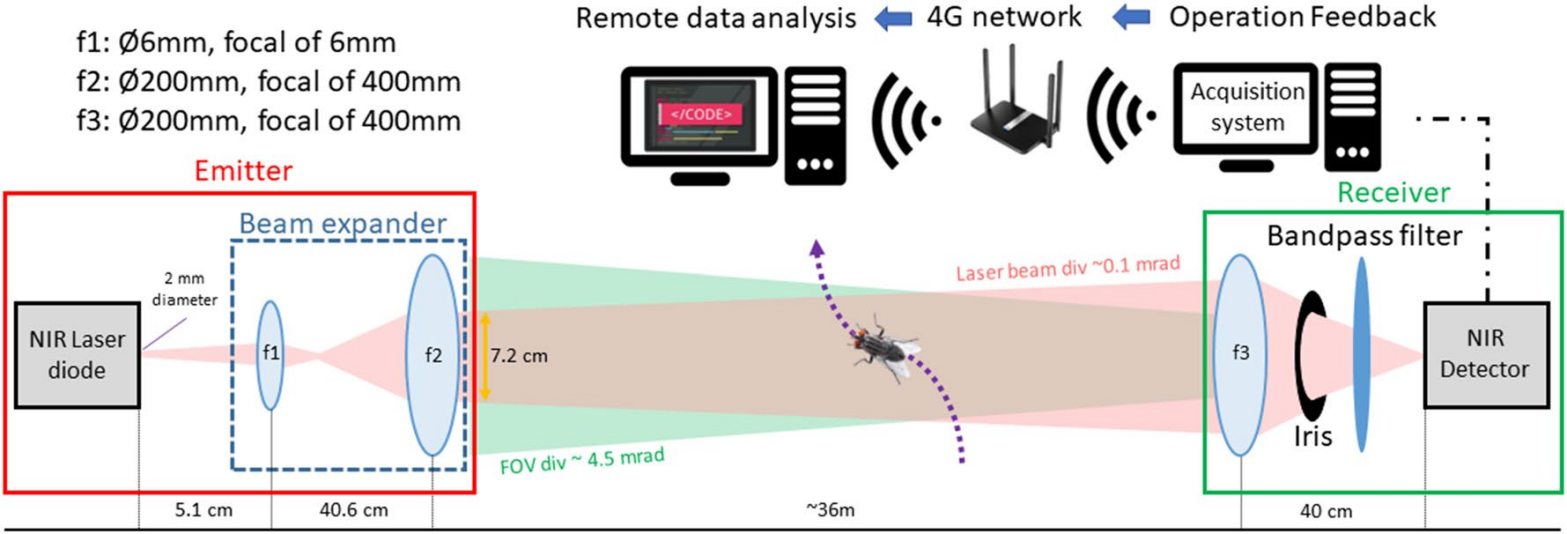
Experimental layout of the Entomological Bistatic Optical Sensor System (eBoss), deployed during the 2021 field campaign. The laser beam is shown in pink and the field of view (FOV) of the receiver in green. The intersection of both defines the probe volume

The eBoss relies on a low-intensity continuous laser diode source (CPS980, Thorlabs, USA) with a peak optical power of 5 mW, operating at 980 nm. Thanks to its low laser power and beam expansion, the system is eye-safe, including for animals, and requires no specific safety measure to operate. As shown in Fig. 1, the laser beam emitted by the laser diode is expanded to Ø72mm using a combination of lenses. The laser beam propagates horizontally over 36 m and about 20 cm above the vegetation which is mowed every other month to prevent any interaction with the laser beam. At the end of the optical path, the light is collected by a converging lens, goes through a bandpass filter and is then focused onto the active area of an amplified, switchable-gain, silicon detector (PDA36A2, Thorlabs, USA). The bandpass filter has a transmission above 95% from 950 to 1000 nm, which allows for drift in laser wavelength caused by the large range of temperature throughout the season. The detector has an effective bandwidth of 90 kHz and a large active area of 3.6 × 3.6 mm. This large active area acts to reduce unwanted fluctuations in the recorded signals caused by small mechanical vibrations on the emitter side. The optical signal is recorded at a sampling frequency of 30,517 Hz over a 5 V range by a 16-bit digitizer (M4i4420- × 8, Spectrum, USA). The equipment was protected from wind, rain, and snow by two tents, one on the emitter and one on the receiver side. The acquisition system was integrated into a regular desktop computer with a screen so that basic check-up of the data could be done on-site. The whole setup consumes approximately 150 W of electrical power, mainly used by the desktop computer, coming from a nearby building. While it was not the direct purpose of this experiment, the whole system could be easily set up to be portable and autonomous: the desktop computer can be replaced by a much smaller integrated acquisition system, this would greatly reduce the power consumption and allow the whole system to be powered by a battery coupled with a small solar panel. Finally, a weatherproof casing would eliminate the need for tents. Operation feedback, such as average signal value and standard deviation, are then sent to the internet data storage (or “cloud”) via a 4G LTE router where they can be monitored remotely by the user to ensure proper operation of the system without the need to physically visit the field location. In addition, a co-located weather station (WS-1002-WIFI, Ambient Weather, USA) was operating continuously to monitor the meteorological conditions such as temperature, rain, wind and UV radiation. Those measurements are saved both locally and in the cloud on the ambient weather network.

**Fig. 2:**
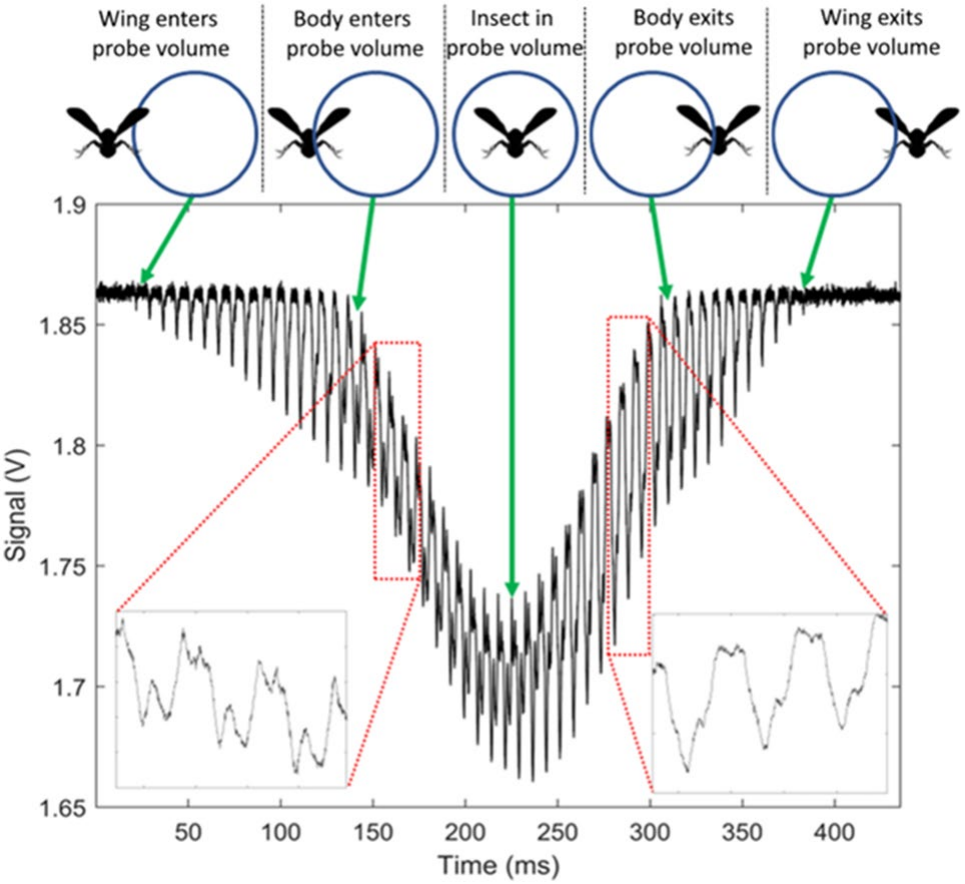
Example of an insect signal as the specimen flies through the probe volume of the eBoss. The bottom left and right corners show a magnification of three full wingbeat cycles of the wing contribution. The repeated patterns (136 Hz) are different when the specimen enters and exits the probe volume, which suggests a possible change in the orientation of the specimen

Figure 2 shows an example of a signal recorded by the eBoss when an insect flies through the probe volume of the system. The probe volume is defined as the intersection between the laser beam and the field of view of the detector, shown in Fig. 1. Considering the value of divergences, the probe volume is almost identical to the volume covered by the laser beam. In the absence of any target in the probe volume, the recorded signal is a constant voltage corresponding to the flux of photons received by the detector. This baseline value can change by a few % over the course of a few hours due to variations in sunlight, change in optical extinction of the probed air or small drift in laser power.

Whenever an insect flies through the probe volume, the signal decreases as the target attenuates the optical intensity through scattering, diffraction, and absorption. Unlike the slow baseline variation mentioned above, the change in signal intensity due to the presence of the insect is on a much shorter time scale, in the order of 20 ms to 1 s. When the insect transits through the probe volume (also referred to as an event), the signal displays a Gaussian-like envelope as the insect enters then exits the probe volume, as shown in Fig. 2. This is caused by the spatial profile of the laser beam and the insect’s trajectory through the probe volume. In addition, the signal presents periodic drop in signal amplitude (sharp peaks) which are due to the rapid change in orientation of the wings of the insect during the wingbeat cycle. This feature allows to discriminate between flying insects that display periodicity and non-insect targets such as falling leaf or pollen that do not display any periodicity, as shown in Fig. 6. The periodicity of the change in signal amplitude also allows for the determination of the wingbeat frequency of the specimen by finding its fundamental frequency, which is done by detecting harmonic series in the frequency spectrum. This also allows for the discrimination between the wing and the body contributions to the signal [51].

**Fig. 3:**
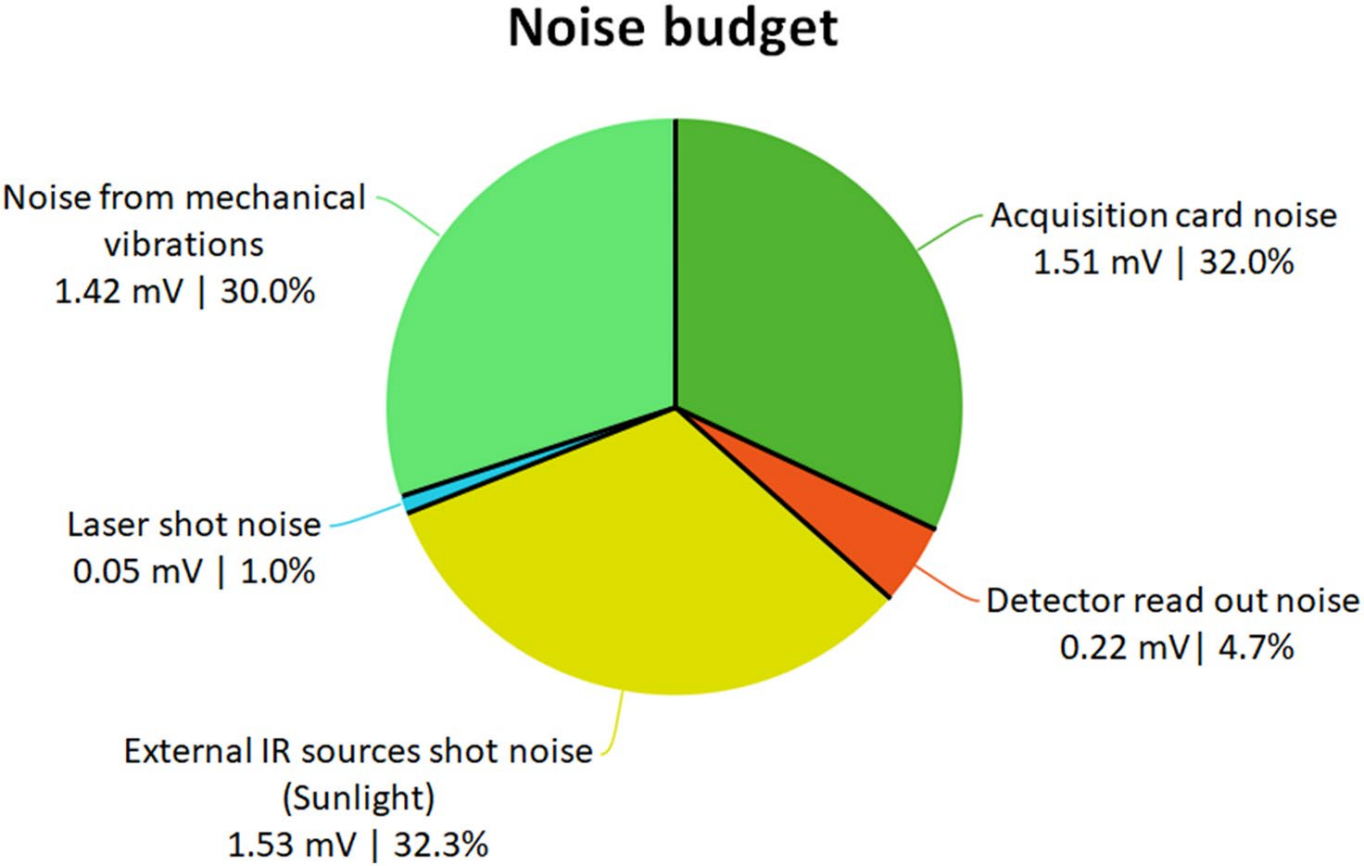
Noise budget of the experiment. Five different sources of noise have been identified and their respective contribution is expressed in mV and in the percentage of total noise. The range of acquisition was between 0 and 5000 mV, spread over the 2^16^ bins of the digitizer. Noise is defined by the standard deviation and the five sources of noise are assumed to be independent and normally distributed

In addition to the baseline variations, the short time scale noise level is the limiting factor of this methodology. Figure 3 shows the noise budget of the experiment. Several areas of improvement can be determined. The three main sources of noise are due to the acquisition card, unwanted IR sources and small mechanical vibrations on the emitter side.

#### Measurement campaign

**Fig. 4:**
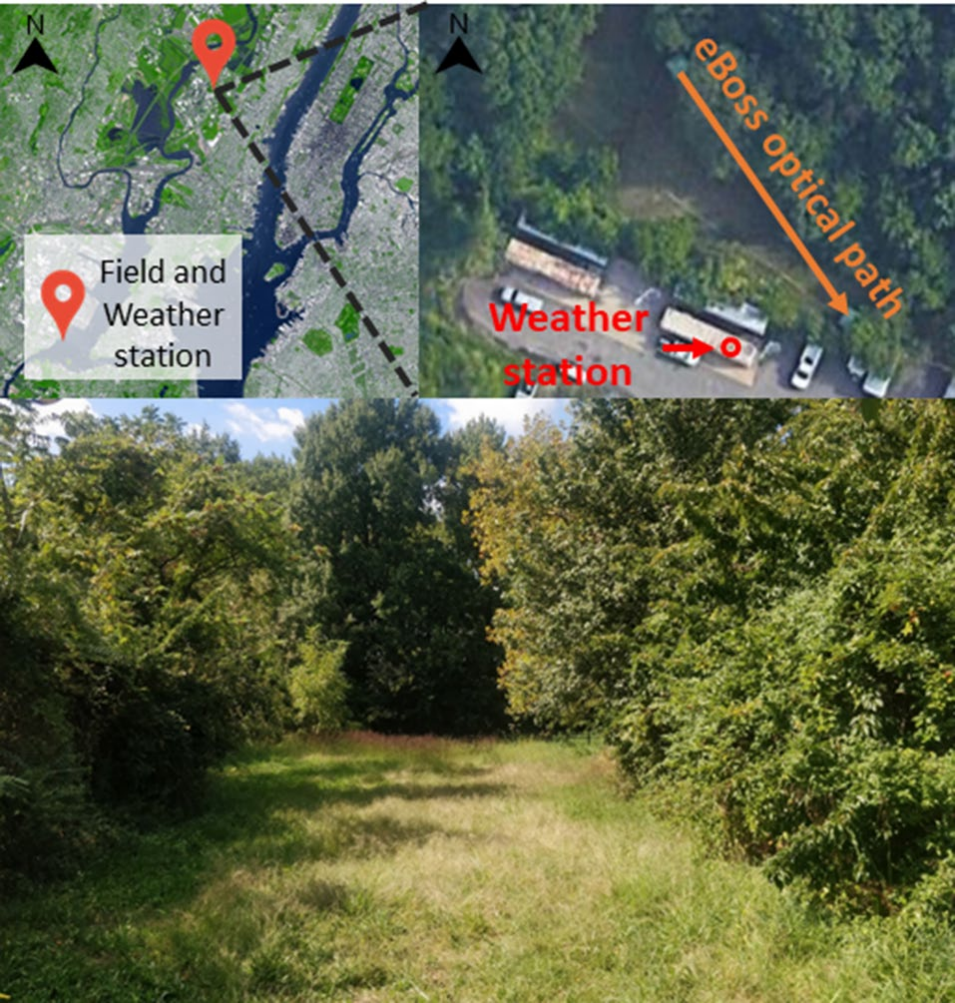
Top left: satellite view of the area. Top right: aerial view of the field location (40°47′09.8″N 74°03′28.1″W) where both green tents used to protect the equipment from rain can be seen. The optical path of the laser beam is indicated by an orange arrow, starting at the emitter side and pointing toward the receiver side. The weather station, red circle, is located on top of a metal container located directly south of the field. Bottom: picture of the field from the receiver side

The measurement campaign took place from July 13 to December 7, 2021. The eBoss was running for 148 consecutive days, with downtime below 6.5% due to temporary shutdowns for alignment verification, routine maintenance and unwanted interruptions. Those few involuntary interruptions of the recording occurred due to the Ida storm of September 2^nd^ that flooded the area and from two power outages. The eBoss was installed in a field in a semi-urban environment, a small patch of green within the city of Secaucus (Hudson County, NJ, USA), see Fig. 4, in the vicinity of one of the world’s largest megalopolises. The field is approximately 40 × 10 m with tall grass bordered by a roughly 1 ha woodlot. A co-located portable weather station was operating next to the field where the measurement campaign took place, as shown in Fig. 4.

### Data analysis

#### Identification of insect signals

**Fig. 5:**
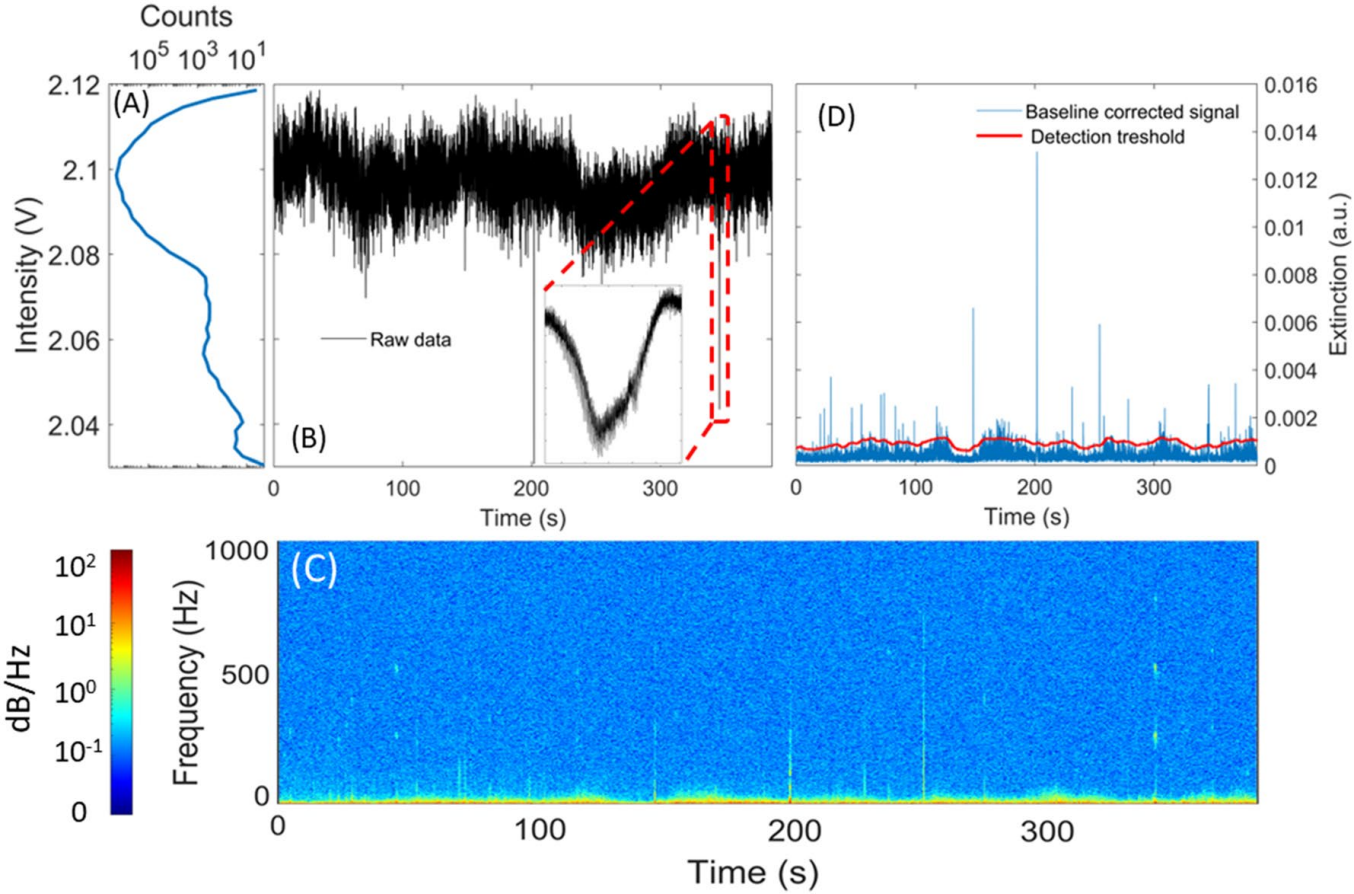
Figure **B** shows 400 s of raw data, as recorded by the acquisition system, with a sampling frequency of 30,517 Hz. A magnification on one of the identified insect events is displayed as an illustration. Figure **A** shows the count per bin of intensity and **C** the spectrogram of the raw data. In the spectrogram, the magnified event stands out and its fundamental frequency as well as its first two harmonics are visible. Figure **D** shows the optical extinction obtained from figure **B**, after the application of a digital bandpass filter [10–900 Hz] and averaging. The red line indicates the detection threshold. Any part of the filtered signal that crosses the threshold is identified as a region of interest and the corresponding raw signal is then extracted for further analysis

Figure 5B displays an example of 400 s of raw data recorded by the eBoss, showing the noise and baseline variations. Figure 5B also shows a magnification on one of the events in the raw time series. Events are characterized by a sudden drop in signal (in the order of a few ms) followed by a sudden increase returning the signal to its baseline value. Events from insects are automatically identified by a two-steps process, the first step consists in identifying a region of interest where an object is likely to have crossed the probe volume, while the second step is used to identify harmonics typical of an insect signal. First, the raw signal is filtered by a 10–900 Hz digital band-pass filter that removes the contributions of slow baseline drift with a period of 100 ms or more and high-frequency noise above 900 Hz. If the extinction of the signal is higher than the detection threshold for more than 0.1 ms, the signal is marked as a region of interest (i.e. a potential event). The detection threshold is defined as the sum of a sliding average of the signal and the sliding standard variation of the signal (red line in Fig. 5D). For both the average and standard deviation, the unweighted sliding windows encompass 5 s of data, which allows for the threshold to follow the slow baseline drifts without being overly sensitive to fast variations that are events. The threshold value is determined by the sum of the sliding average and 2.5 times the sliding standard deviation.

**Fig. 6:**
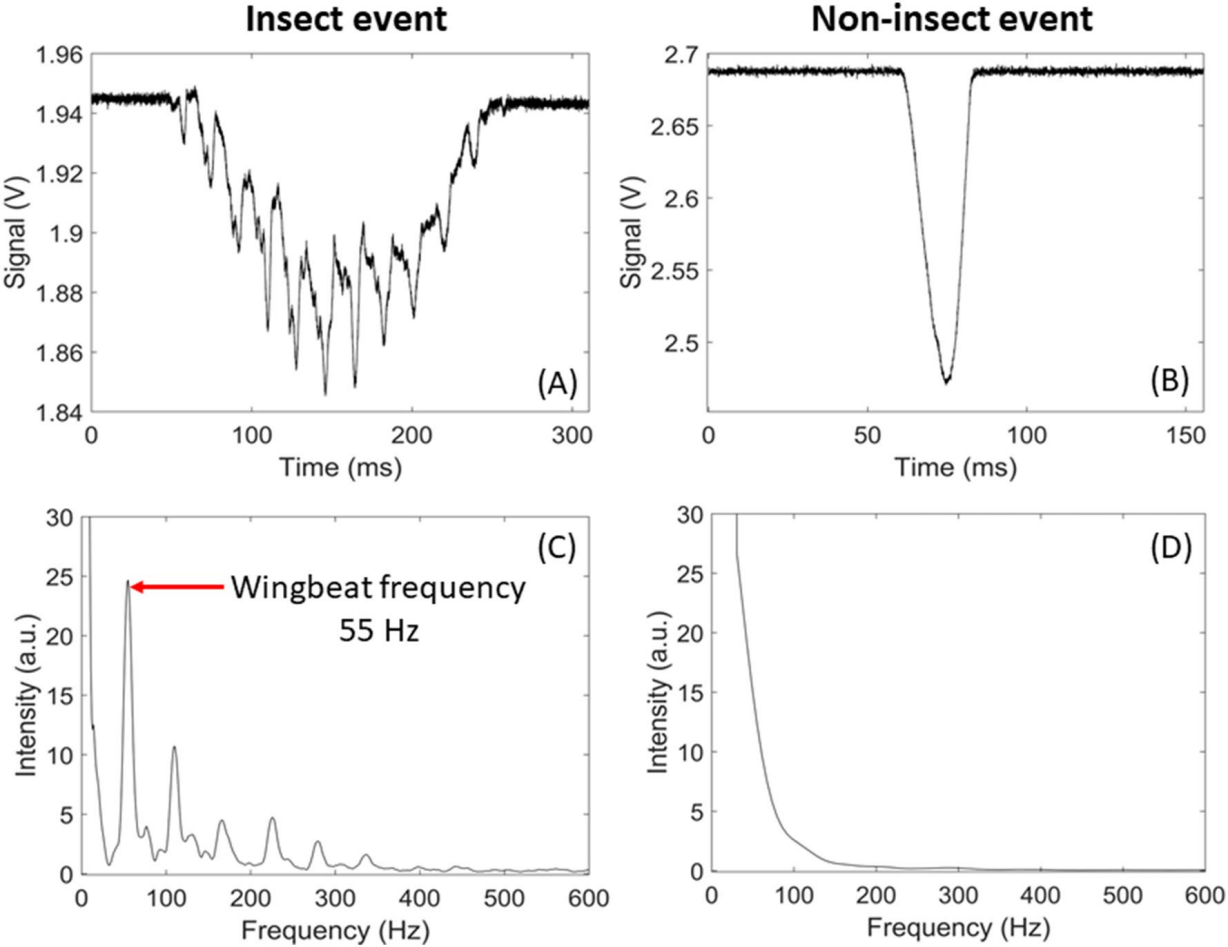
Example of two types of signals and their corresponding frequency analysis. Figure **A** is an example of signal caused by an insect showing clear periodic drop in signal amplitude. Figure **C** shows its associated Fast Fourier Transform, from which the insect wingbeat frequency can be determined. Figure **B** is an example of a signal from a non-insect target crossing the probe volume, which does not have any periodic drop in signal amplitude, as can be seen on its Fast Fourier Transform (Figure **D**)

This method effectively identifies all possible events in the raw signal that have frequency components in the 10–900 Hz range. In practice, a non-negligible number of false events (i.e., not caused by a flying insect) are selected as the region of interest. Therefore, the second step focuses on identifying harmonic components within a dynamic frequency range: the lower limit is defined from the duration of the event so that at least two full wingbeat periods can be found in the signal, the higher limit is set at a fix value at 2 kHz. Well-defined harmonics are typical of a signal originating from flying insects and not simply from a leaf, pollen or large aerosols passing through the laser beam. While effective, this method has two main limitations: the first is that small insects for which the optical extinction is too low to cross the detection threshold will not be detected. The second is for events that present no clear periodic signal, this can happen if the wings do not fully enter the probed volume, or if the time of transit is too short to observe multiple wingbeat cycles. Figure 6 presents an example of two recorded events showing one from an insect with the periodic amplitude modulations typical of an insect (Fig. 6A and C) and one from a non-insect object (Fig. 6B and D).

#### Extinction cross sections

**Fig. 7:**
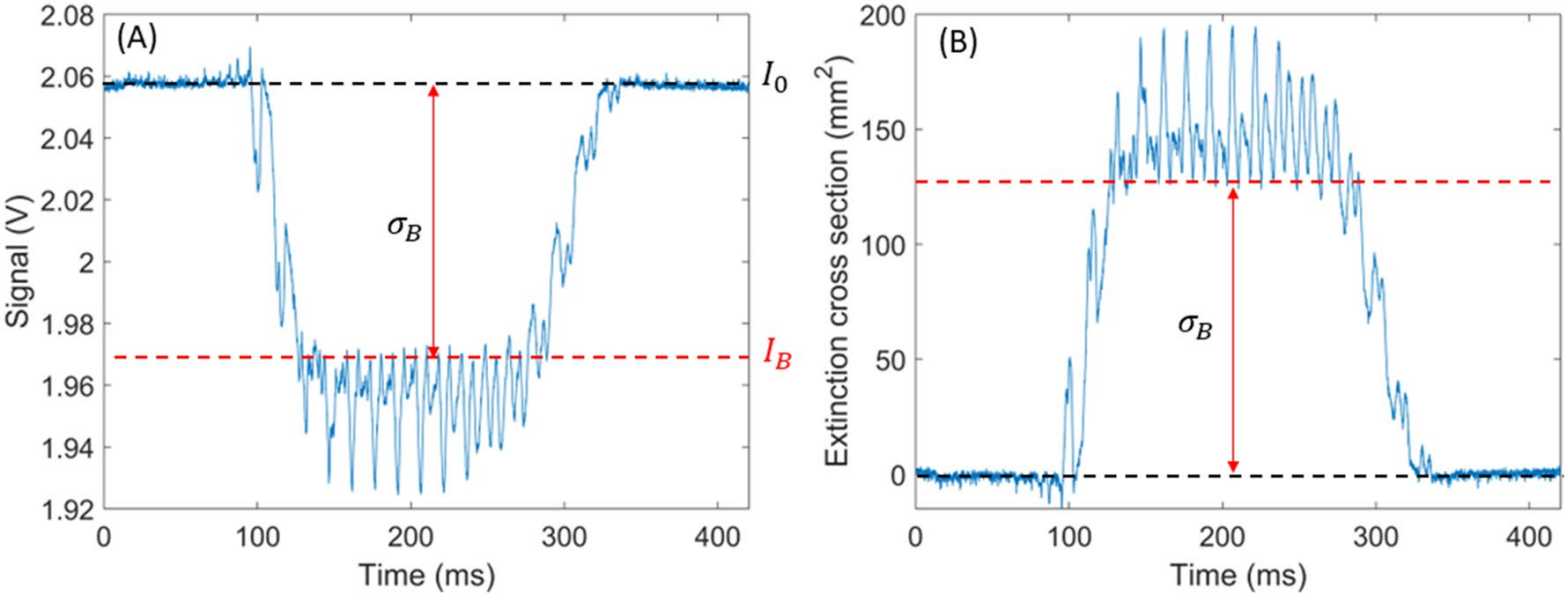
Figure **A** shows a signal due to the crossing of an insect through the probe volume of the eBoss. The red arrow indicates the amplitude of the signal decrease that is due to the body of the insect. ${I}_{0}$ is the value of the baseline and ${I}_{\mathrm{B}}$ is the value taken by the signal when the body of the insect is completely in the center of the probe volume. Figure **B** shows the same event after conversion in terms of extinction cross section, using Eq. (1)

Because the cross section of the probe volume is known, the extinction cross section of the insect expressed in mm^2^ can be derived from the drop in voltage measured by the instrument. The iris placed in front of the detector (Fig. 1) allows to remove the wings of the Gaussian profile of the laser beam from the probe volume. As a result, the energy density of the laser beam can be assumed to be constant within the probe volume (also sometimes referred to as the “flat top approximation”). Consequently, the insect extinction cross section can be derived by Eq. (1):

1 $${\sigma }_{\mathrm{B}}={\sigma }_{\mathrm{pv}} \cdot \frac{{I}_{0}-{I}_{\mathrm{B}}}{{I}_{0}}$$

where ${I}_{0}$ is the intensity of the signal before the transit event and ${I}_{B}$ the value of the maximal signal decrease due to the insect body, as shown in Fig. 7. The cross section of the probe volume is denoted ${\sigma }_{\mathrm{pv}}$ and the extinction cross section of the insect body ${\sigma }_{\mathrm{B}}$, both expressed in mm^2^. The validity of this approach has been confirmed by dropping opaque chrome steel spheres of known diameters through the probe volume, showing a maximum error in retrieved extinction cross section of 6%.

#### Insect mass estimation

When considering an insect $n$, the extinction cross section of its body ${\sigma }_{n}$ is a function of the geometrical cross section of the insect body ${A}_{n}$ and the quasi-ballistic transmittance of the insect ${T}_{\mathrm{b},n}$, as described by Eq. (2). Quasi-ballistic photons being the photons that are transmitted and that reach the active area of the photodetector. Those photons are the ones that are not absorbed, and that undergoes a sufficiently small amount of scattering or diffraction so that their direction of propagation remains within the solid angle of detection of the photodetector.

2 $${\sigma }_{n}= (1-{T}_{\mathrm{b},n})\cdot {A}_{n}$$

The volume of the insect can be estimated from its geometrical cross-section using Eq. (3):

3 $${V}_{n}={K}_{n} \cdot {A}_{n}^{3/2}$$

where the volume in the insect ${V}_{n}$ is expressed as a function of the geometrical cross section of its body ${A}_{n}$ and as a function of ${K}_{n}$ a proportionality factor. ${K}_{n}$ is a unitless factor as long as ${V}_{n}$ and ${A}_{n}^{3/2}$ are both expressed with the same volumetric unit.

The mass of an insect $n$ is denoted ${m}_{n}$ and is equal to its volume ${V}_{n}$ times its volumetric mass density ${\rho }_{n}$, see Eq. (4). The volumetric mass density of insects may vary from one species to the next. However, Kühsel et al. [52] showed the relationship between an insect volume and its mass to be mainly linear, *R*^2^ = 0.92, across 113 insect species, indicating that considering the volumetric mass density of insects as constant across species is a reasonable approximation.

4 $${m}_{n}={\rho }_{n} \cdot {V}_{n}$$

By combining Eqs. (2), (3) and (4) and by applying the aforementioned approximations (where ${T}_{\mathrm{b},n}$, ${K}_{n}$ and ${\rho }_{n}$ are considered constant across all insect species), the following relationship is derived:

5 $${m}_{n}= \rho \cdot K \cdot {\left(\frac{{\sigma }_{n}}{(1-{T}_{\mathrm{b}})}\right)}^{3/2}= \eta \cdot {\sigma }_{n}^{3/2}$$

The factor $\eta$ takes into account the difference between geometrical and extinction cross sections, the relationship between surface and volume and the volumetric mass density of insects. While $\eta$ is expressed in the unit of mass per unit volume, it is important to note that it is not equal to the volumetric mass density of insects. To evaluate coefficient $\eta$, a laboratory eBoss prototype was used to retrieve the body extinction cross section of insects of known weight. Below is the list of insect species used for this experiment:

- Mosquitoes (both *Culex quinquefasciatus* and *Aedes aegypti*): 122 specimens.
- Flies (*Musca domestica*): 50 specimens.
- Bees (*Osmia lignaria*): 6 specimens.
- Wasps (*Vespula maculifrons*): 5 specimens.
- Bumble bees (*Bombus bimaculatus*): 4 specimens.

The number of specimens varies as some where available in large quantities by rearing them while others were captured in the field. Insects were weighed immediately upon their death as well as post desiccation using a 0.1 mg precision scale, to determine their wet and dry mass.

#### Flying insect volumetric biomass density estimation

Using Eq. (5), the mass of every flying insect that crosses the probe volume is estimated. This allows for the determination of the mass of flying insects per unit of volume, referred to as biomass density in this contribution, denoted ${\rho }_{\mathrm{b}}$, which can be calculated for both wet and dry insect mass using Eq. (6):

6 $${\rho }_{\mathrm{b}}=\frac{{\sum }_{n=1}^{N}\frac{{\Delta t}_{n}}{{t}_{\mathrm{tot}}}\cdot {m}_{n}}{\Delta V}$$

This equation is derived from a statistical approach detailed in earlier work [53] where the aerial density (insects/m^3^) is determined but modified to be expressed in terms of biomass density instead. $N$ is the number of events observed during the time period ${t}_{tot}$. For each event, the mass ${m}_{n}$ is retrieved using the methodology described in the previous section, while the transit time ${\Delta t}_{n}$ represents the duration during which the insect was within the probe volume of the eBoss, see [50, 53] for more details. As shown by Eq. (6), the biomass density ${\rho }_{\mathrm{b}}$ is normalized by the volume of air probed by the instrument $\Delta V$ so that it is expressed in mg/m^3^.

The time resolution of the retrieved biomass density is defined by ${t}_{\mathrm{tot}}$, which can be set to days, weeks or months to observe the long-term evolution of the biomass density or set to minutes or hours to observe daily change in biomass density. As thoroughly discussed in previous work [50, 53], the number of observed events, and therefore the retrieved biomass density, is subject to stochastic fluctuations. For this reason, there is a tradeoff between time resolution and uncertainty: small time scale, in the hour or minute range, may present significant statistical fluctuations on the retrieved biomass density while a longer time resolution may provide more robust results but these will be an average over any density variations that occurred during the sampling period.

## Results

### Laboratory results for insect mass estimation

**Fig. 8:**
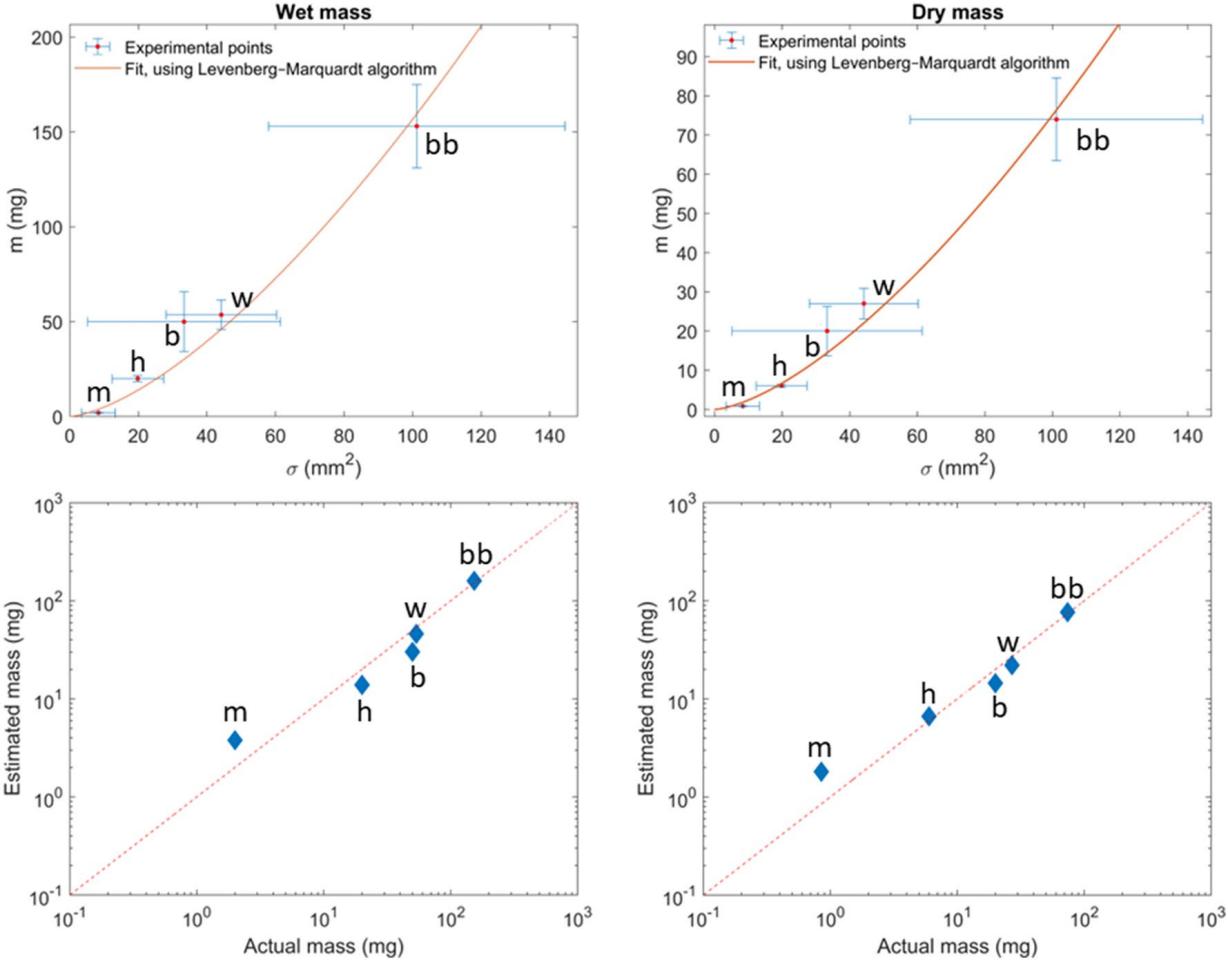
Figure **A** and **B** display the result of the fit of Eq. (5) for the wet and dry mass, respectively. Error bars represent the standard deviation. Figure **C** and **D** display the results of the estimated mass (respectfully wet and dry) using the results of the previous fit, Eqs. (7) and (8), as a function of the actual mass measured with a 0.1 mg precision scale. Each insect group is identified by a unique marker, m for Culicidae, h for Musca domestica, b for Osmia lignaria, w for Vespula maculifrons and bb for Bombus bimaculatus

As presented in Sect. 2.2.3, Eq. (5) allows for the retrieval of the mass of an individual insect using the measured extinction cross section of the specimen and the coefficient $\upeta$. A fit of Eq. (5) provides an estimation of the insect mass $m$ from their body extinction cross section $\sigma$ (Fig. 8). In terms of percent error between predicted and actual mass, the worst prediction is for the dry mass of mosquitoes with a 113% percent relative error. However, the absolute error is only 1 mg, meaning that the dry mass of mosquitoes tends to be overestimated by 1 mg. On the other hand, the best prediction in terms of percent error is for the dry mass of bumblebees with only a 3.2% difference, due to an average overestimation of 2.4 mg.

Using a Levenberg–Marquardt algorithm [54, 55] to fit Eq. (5), the values of $\upeta$ can be estimated for both the wet and dry insect mass. This fit allows for the determination of the relation between mass and extinction cross section of the insect body, as shown by Eqs. (7) and (8).

7 $${m}_{\mathrm{wet}}= 0.157\cdot {\upsigma }^{3/2} {R}_{\mathrm{adj}}^{2}=96\mathrm{\%}$$

8 $${m}_{\mathrm{dry}}= 0.075 \cdot {\upsigma }^{3/2} {R}_{\mathrm{adj}}^{2}=98\mathrm{\%}$$

where $m$ is the estimated mass in mg (either dry or wet), $\sigma$ the insect body extinction cross section in mm^2^. As such, the value of $\eta$ is expressed in mg/m^3^ in both aforementioned equations.

### Seasonal variation of flying insect biomass density

**Fig. 9:**
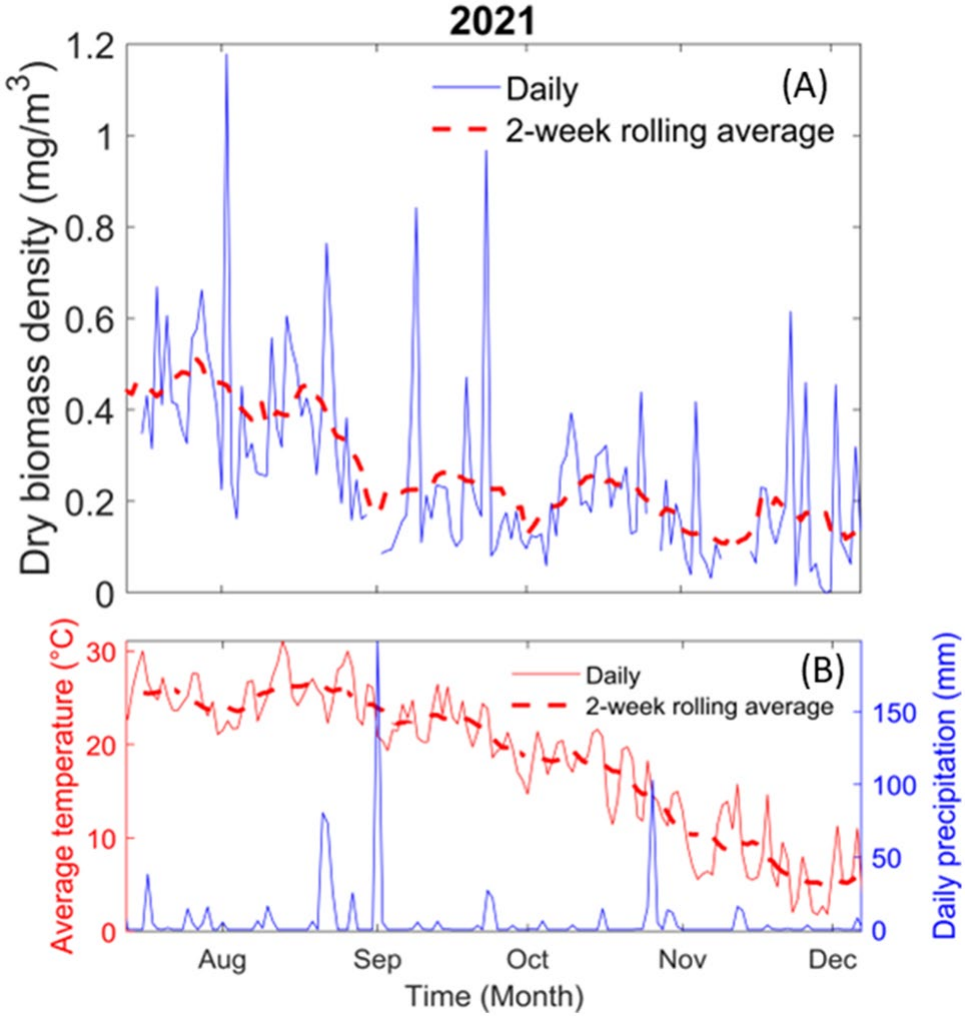
In figure **A**, the solid blue line represents the daily flying insect biomass density per meter cube of air and the red dashed line its 2-week rolling average. The biomass density, expressed in mg/m^3^, is the dry biomass of the insect. In figure **B** the solid red line represents the daily average temperature and the dashed red line its 2-week rolling average. The solid blue line represents the daily precipitation

As shown in Eq. (6), the dry biomass density can be retrieved on a user-defined time resolution. In Fig. 9, the sliding two weeks average over a one-day time resolution has been presented as it reduces stochastic fluctuations possibly caused by changing atmospheric conditions and by the statistical nature of the process. In the rest of the publication, only the results of dry biomass density will be presented. The dry biomass density was chosen over the wet biomass density for several reasons. The wet biomass includes the insect water content which may not be relevant when evaluating the quantity of food availability for predators. Moreover, the dry biomass is more easily compared with the gold standard results of insect traps, which often only provided dry insect mass from laboratory measurements. In addition, the dry mass estimation, from our described methodology, is more precise than the wet mass estimation.

As shown in Fig. 9, the flying insect biomass density is following a downward trend as time passes. This result was expected as the population of several insect species are known to decrease with decreasing temperatures. Interestingly, however, the observed biomass density does not drop to 0 mg/m^3^, even in early December when the campaign stopped. Despite the low temperature, some insects remain active in December but only during the warmest hours of the day and very little activity is seen at night when temperatures are the lowest (see the following section and Fig. 11).

Figure 9 shows several fluctuations in the total biomass density which may be an indication of the emergence and disappearance of seasonal species or a shift in insect behavior. From mid-July to late August, the flying insect biomass density is at its highest values, oscillating around 0.5 mg/m^3^. During this time, the max value of biomass density was around 1.2 mg/m^3^ when considered over an entire day. On the other hand, the minimum of flying insect activity takes place from the end of November to December when the biomass density varies around 0.1 mg/m^3^ when considered over an entire day. Despite the average daily temperature dropping near 3 °C during December, there was continuous flying insect activity.

**Fig. 10:**
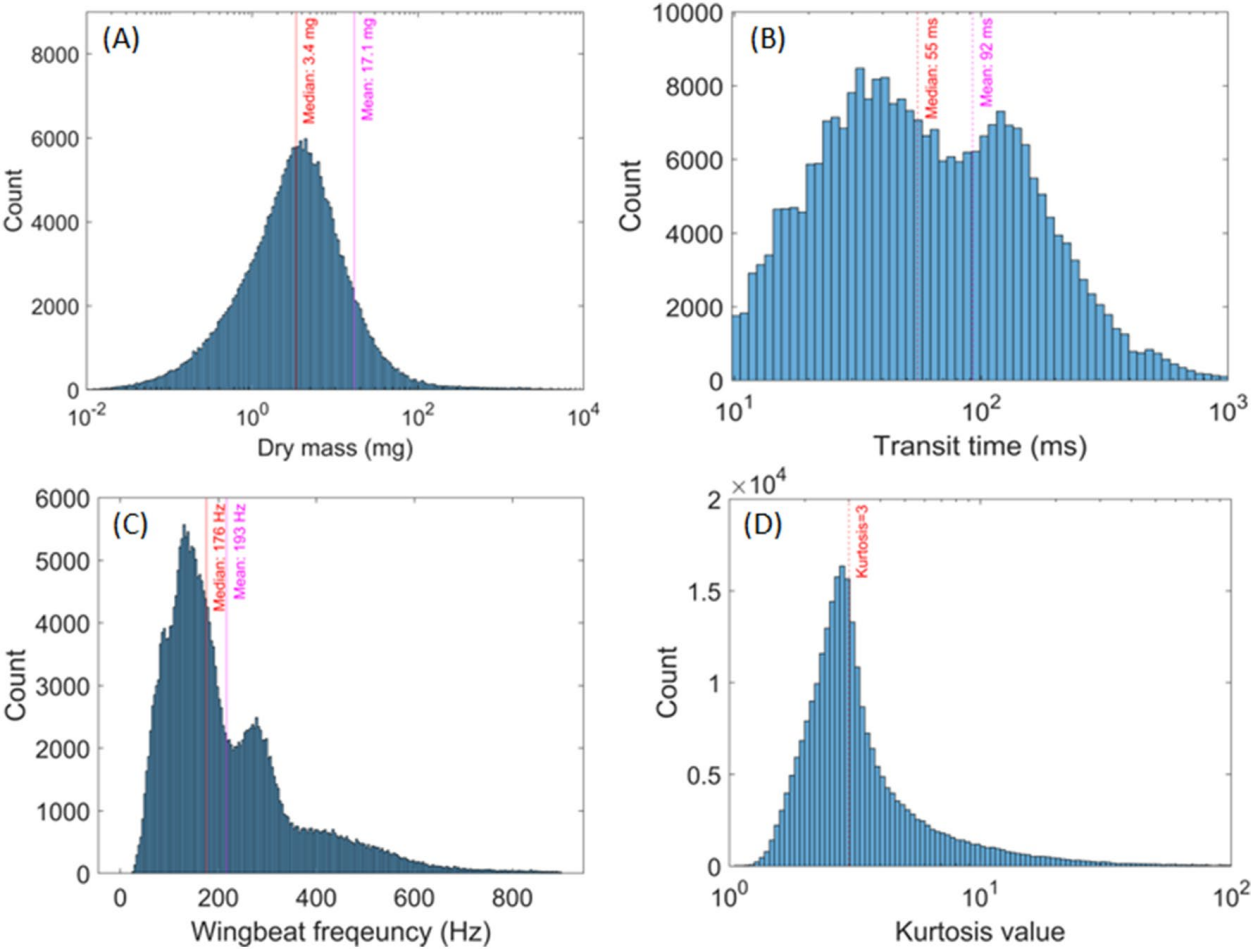
Figure **A** displays the distribution of the retrieved mass of insects, using Eq. (8). The median and mean value are displayed in red and magenta, respectively. Figure **B** shows the distribution of the transit times of insets, i.e., the duration of their transit through the probe volume, which is related to the insect flight velocity. The median and mean value are displayed in red and magenta, respectively. Every event with a transit time lower than 10 ms were systematically removed (hard cut-off at 10 ms). Figure **C** illustrates the wingbeat frequency distribution of insects. The median and mean value are displayed in red and magenta, respectively. Figure **D** displays the kurtosis value distribution of every event, which provides information on the shape of the signal. A kurtosis value equal to three, displayed in red, correspond to a Gaussian shape

In addition to the seasonal variation of the biomass density, the mass of each of the 262,870 insect events observed throughout the season can be estimated. This provides information on the mass distribution of insects on the field during 2021. The dry mass distribution can be seen in Fig. 10A, showing the average and median values. Low masses in the range of 0.1 mg are observed due to insects only partially entering the field of view of the sensors, resulting in an underestimation of the mass of the insect. Between July and December 2021, the average dry mass of insects observed was 17.1 mg and the median value was 3.4 mg. Moreover, the distribution of the transit time and wingbeat frequency of the insect events are presented in Fig. 10B and C, respectively. Finally, in Fig. 10D, the distribution of the kurtosis value of every event is presented, which informs on the shape of the event, a kurtosis value of three corresponding to a Gaussian shape.

### Daily variation of flying insect biomass density

**Fig. 11:**
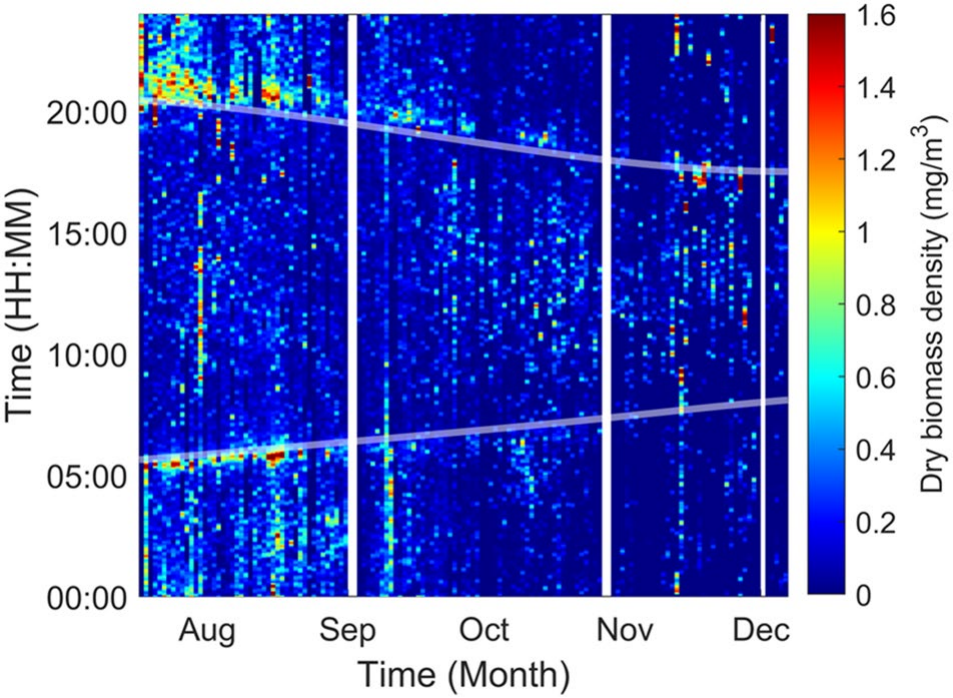
Dry biomass density estimation in function of the time-of-day with a 1-min resolution and over the entire measurement campaign of the eBoss. Maximal values of biomass density were artificially capped at 1.6 mg/m^3^ to improve the color plot contrast. The white lines indicate periods during which the system was offline. The semi-transparent lines indicate the civil sunrise and sunset time at the field location

**Fig. 12:**
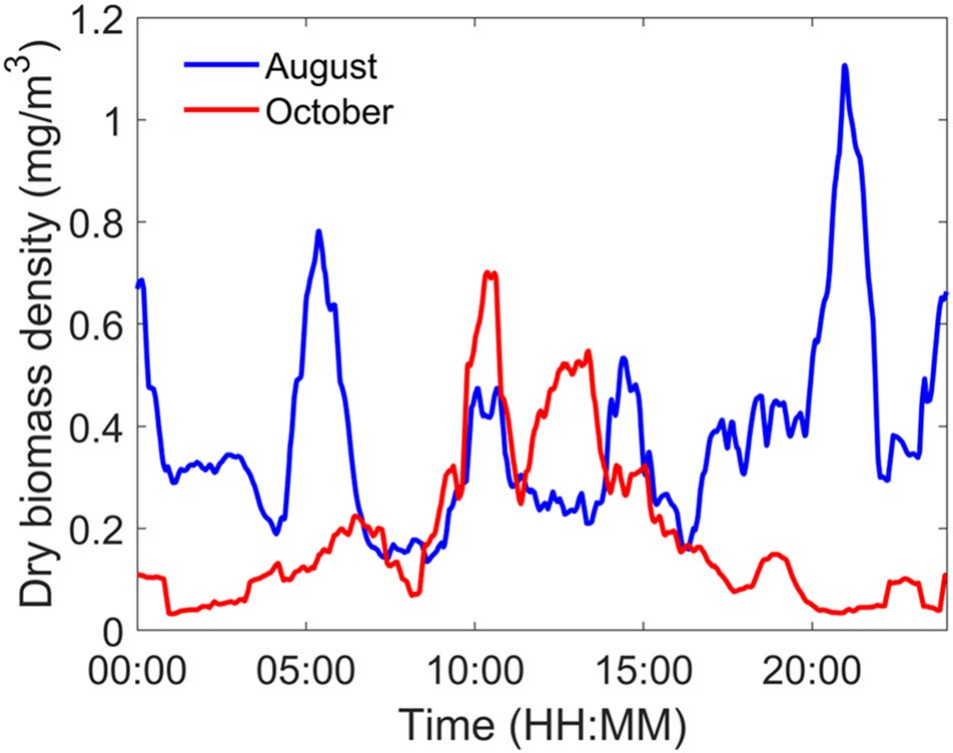
Dry biomass density over 24 h. Blue and red line represent the average daily variation of the dry biomass density for the month of August and October, respectively. The presented results are the sixty minutes rolling average of the biomass density, averaged over the entire month

The eBoss allows for the study of biomass density on a much shorter time resolution than the weekly average, down to the minute range, as shown in Figs. 11 and 12. This allows for the refined study of insect activity and the observation of how biomass density varies during a single day as well as seasonal variation.

The biomass density of flying insects varies throughout the day and is overall greater during sunrise and sunset where an increase in biomass density can be observed. As shown in Fig. 11, the biomass density goes to higher values than when considered over longer periods of time (Fig. 8), above 1.6 mg/m^3^, in particular around sunset and sunrise time. The civil sunset and sunrise times are indicated by the semi-transparent lines in Fig. 11. These results were expected as the behavior of several insect species is driven by sunlight [56–58], furthermore it is coherent with previous observations done at this location [50].

The daily biomass density distribution over 24 h is presented in Fig. 12 for both the month of August (blue) and October (red). In August, there are two peaks of activity around 5:30 am and 9 pm, corresponding to sunrise and sunset time respectively, with strong residual activity at night. On the contrary, during the typical day of October, the dominant period of biomass density is no longer centered around sunset and sunrise but around noon with much less activity at night. This can be the consequence of a change in insect populations or behavior between August and October. Insects being cold blooded, their mobility can be reduced with colder temperatures, favorizing activity during the day, as observed in October. Such information can be helpful for entomologists to infer the presence or absence of some insect species by comparing those results to known insect circadian rhythms. This can also be combined with the observed mass of the insects, and their wingbeat frequency, providing several parameters for species identification purposes.

## Discussion and conclusion

This study presents continuous measurements of the flying insect biomass density using an Entomological Bistatic Optical Sensor System (eBoss) and obtained during a five-month-long campaign. The strength of this approach mainly lies in its ability to estimate the body masses of insects transiting through the field of view of the sensor from which quantitative estimates of aerial biomass densities can be retrieved in SI units (mg/m^3^). The data are collected with a temporal resolution in the minute range, allowing to study daily variations in biomass density as well offering an alternative to traps with much lower temporal resolution. Because the system was designed to require only limited supervision and manageable amount of data (about 4 GByte/day), it can operate for extended periods of time, making the monitoring of aerial biomass density possible over months, like presented here, and possibly years for a low price and with limited manpower. We believe that such features could potentially provide a solution to increase the very low number of long-term studies about insect biomass currently available. Finally, these measurements are obtained without capturing and killing insects, which may be a crucial aspect when targeted at endangered species.

This experiment also reveals some of the weakness of this approach. Because insects can sometimes only partially enter the field of view of the sensor, the retrieved insect mass can possibly be underestimated, see Fig. 10A, especially for large insects that are more likely to enter only partially the laser beam. Increasing the laser beam diameter would reduce the probability of underestimating insect masses, on top of providing a larger volume of probed air, but at the cost of lower sensitivity to small insects that could become undetected due to low SNR. Although, we believe that significant improvements in SNR can be achieved with a new updated sensor design that is currently being tested. In addition, the identification of the insect species remains much more difficult, as the insect is never captured. However, this issue may be solved as more and more progress are made toward the identification of insects using machine learning classifiers from their retrieved wingbeat frequencies, wing and body optical cross-sections, or the timber of the wing signal using Mel-frequency cepstral coefficients [57].

This field experiment followed one done in 2020 and presented by Genoud et al. [50]. In this experiment, the optical sensor used a backscattered configuration, similar to a lidar, as opposed to the extinction configuration here. While it is beyond the scope of this article to provide a thorough comparison between the two systems, we can briefly note that the eBoss has the great advantage of being eye-safe as it requires only a few milliwatts of optical power, compared to the few watts necessary in backscattered configuration, making it cheaper as well. This may be key for long-term and unsupervised deployments of multiple sensors in the future. However, the eBoss is a bistatic sensor with an emitter and receiver, which make its deployment more complex and limits where the instrument can be deployed: it can hardly be pointing vertically or above inaccessible terrains or bodies of water, as opposed to the backscattered configuration that is much more versatile in that respect.

## Data Availability

The datasets generated and/or analyzed during the current study are available from the corresponding author on reasonable request.
